# StackDPP: Stacking-Based Explainable Classifier for Depression Prediction and Finding the Risk Factors among Clinicians

**DOI:** 10.3390/bioengineering10070858

**Published:** 2023-07-20

**Authors:** Fahad Ahmed Al-Zahrani, Lway Faisal Abdulrazak, Md Mamun Ali, Md Nazrul Islam, Kawsar Ahmed

**Affiliations:** 1 Computer Engineering Department, Umm Al-Qura University, Mecca 24381, Saudi Arabia; 2 Department of Computer Science, Cihan University Sulaimaniya, Sulaimaniya 46001, Iraq; lway.faisal@sulicihan.edu.krd; 3 Department of Software Engineering (SWE), Daffodil International University (DIU), Sukrabad, Dhaka 1207, Bangladesh; mamun35-274@diu.edu.bd; 4 Division of Biomedical Engineering, University of Saskatchewan, 57 Campus Drive, Saskatoon, SK S7N 5A9, Canada; 5 Department of Community Health & Epidemiology, University of Saskatchewan, Saskatoon, SK S7N 5E5, Canada; nazrul.i@usask.ca; 6 Department of Electrical and Computer Engineering, University of Saskatchewan, 57 Campus Drive, Saskatoon, SK S7N 5A9, Canada; 7 Group of Biophotomatiχ, Department of Information and Communication Technology, Mawlana Bhashani Science and Technology University, Santosh, Tangail 1902, Bangladesh

**Keywords:** mental health, depression model, risk factors, StackDPP, physicians in Bangladesh

## Abstract

Mental health is a major concern for all classes of people, but especially physicians in the present world. A challenging task is to identify the significant risk factors that are responsible for depression among physicians. To address this issue, the study aimed to build a machine learning-based predictive model that will be capable of predicting depression levels and finding associated risk factors. A raw dataset was collected to conduct this study and preprocessed as necessary. Then, the dataset was divided into 10 sub-datasets to determine the best possible set of attributes to predict depression. Seven different classification algorithms, KNN, DT, LGBM, GB, RF, ETC, and StackDPP, were applied to all the sub-datasets. StackDPP is a stacking-based ensemble classifier, which is proposed in this study. It was found that StackDPP outperformed on all the datasets. The findings indicate that the StackDPP with the sub-dataset with all the attributes gained the highest accuracy (0.962581), and the top 20 attributes were enough to gain 0.96129 accuracy by StackDPP, which was close to the performance of the dataset with all the attributes. In addition, risk factors were analyzed in this study to reveal the most significant risk factors that are responsible for depression among physicians. The findings of the study indicate that the proposed model is highly capable of predicting the level of depression, along with finding the most significant risk factors. The study will enable mental health professionals and psychiatrists to decide on treatment and therapy for physicians by analyzing the depression level and finding the most significant risk factors.

## 1. Introduction

Depression (major depressive disorder) is a widespread and significant medical ailment that harms the quality of life, thought, and behavior [1]. It is, luckily, curable. Depression leads to a feeling of melancholy and loss of interest or pleasure in previously appreciated activities [2]. It can cause a wide range of mental and physical difficulties and a reduction in a person’s ability to perform at work and home [3]. According to a report by the World Bank, nearly 1 billion people experience mental disorders or depression, and around 75% of them do not take any clinical initiative for mental health [4]. Mental health and depression result in suicidal cases when a mental health condition reaches intolerable levels. The World Health Organization (WHO) reports that a person commits suicide every 40 s, and 77% of suicidal incidences occur in lower- and middle-income countries [5]. About 1.3% of total deaths happened by suicide making it the 17th leading cause of death. The suicide rate among physicians has been reported to be between two and five times that of a normal community [6]. It has been found that the rates of mental disorders can double during different crises, such as the recent COVID-19 pandemic, natural disasters, civil wars, and so on [7]. The mental health of nearly 59% of the total population has been affected by the COVID-19 pandemic in the United States [8]. Depression is anticipated to place a greater cost on nations over the next 10 years than any other ailment [4]. Doctors have greater rates of anxiety and depression than the overall population and other professional groups in most healthcare systems (whether privately or publicly supported) and across all ages, genders, specializations, and statuses [9]. This seems contradictory considering that doctors enjoy a plethora of ostensibly protective characteristics, such as career and financial stability, high reputation, and usually fulfilling employment [10]. Doctors, like everyone else, are vulnerable to the hazards linked with genetic predisposition, early traumatic life events, later loss, diseases, or relationship failures [11]. Physicians’ mental health is also connected to how physicians are addressed, how they manage psychologically, special challenges associated with their employment, and a structure in which doctors with psychological problems are dealt with in an adversarial rather than treatment-focused manner [12]. Working with COVID-19 patients has been another significant risk factor for physicians during the COVID-19 pandemic.

In Bangladesh, almost 7 million people experience depression and mental health issues, according to a recent study [13]. Another study conducted in 2021 in Bangladesh states that anxiety and depression during COVID-19 were 69.5%, and 39.5%, respectively, for less severe symptomology (at least borderline abnormal), and 41.% and 15.7% for more severe (at least abnormal) symptomology among healthcare workers [14]. Another study conducted in 2021 reports that 55.3% of medical professionals were experiencing depression, whereas 5.2% of medical professionals were facing anxiety, a preliminary stage of depression, in Bangladesh [15]. It is also stated by the study that Bangladeshi female clinicians were found to be more stressed than male physicians. Almost 52% of medical professionals start their career having depressive symptoms since studying at medical college [16]. This report indicates that early-career medical professionals also experience depression from the very beginning of their professional life. It is hazardous for a nation unless the mental health of every medical professional is assessed and taken care of.

It is crucial to take care of physicians’ mental health to keep a nation healthy because they are continuously working on the frontline in situations such as COVID-19. According to one study, having a mental disease with co-morbidities can cut life expectancy by around 20 years [17]. To ensure the continuity of health treatment, medical organizations should prioritize the psychological health of these frontline workers [18]. It is critical to establish the mental health state of Bangladeshi physicians to assist the physician community. This is particularly important because the mental health of physicians impacts not only themselves but also their professional performance and hence the care of patients. It has been observed that depressed physicians make six times more treatment errors than healthy professionals [19]. Therefore, it is crucial to keep physicians mentally healthy. From the prior discussion, it is clear that keeping physicians mentally healthy is the first and foremost step to ensuring quality treatment. To ensure healthy mental health, mental health screening tests are required, which are expensive, time-consuming, and unavailable everywhere. In addition, mental health professionals need to know the level of depression for primary treatment and suggestions. An automated screening test device could solve this issue by detecting the level of depression in a physician. Because nowadays, the machine learning approach is playing a vital role in the detection and prediction of different diseases, it could be a potential solution to detecting the level of depression based on some of these attributes.

In recent years, some studies have been conducted to diagnose and detect depressive symptoms using audio from daily conversations using a machine learning approach [20,21,22,23]. More studies have been conducted to diagnose and detect depression by observing the social media activities of a patient [24,25,26,27]. Zhou et al. in 2022 conducted a study to reveal depressive symptoms among physicians, although only for Chinese during COVID-19 [28] The study was limited to only in COVID-19 pandemic because they mostly focused on COVID-19-related issues. However, there are some professional issues for a physician, which are not found in social media activities. In addition, these professional issues are significantly responsible for depression in a physician, which some recent studies have shown [29]. Most importantly, all of these methods are time-consuming, because audio conversation observation takes some days to diagnose, and tracking social media is time-consuming for a physician. For physicians, an automated device is required, which will diagnose and detect the level of depression in a moment. From this perspective, the study aimed to employ a machine learning approach to find potential attributes which would enable the diagnosis and detection of depression levels and build a machine learning model to diagnose and detect the level of depression of a physician within a moment. The proposed system would be less time-consuming, more cost-effective, and more efficient in performance. The contributions of this study are summarized as follows:

1. Building an efficient stacking-based ensemble classifier, which will be able to diagnose the mental health stage of clinicians with higher accuracy.
2. Finding the best subset of features that are the most significant and risky for clinicians.
3. Analyzing the most significant risk factors for the mental health of clinicians.
4. Investigating whether only one group of attributes, such as only PHQ-related features, sociodemographic, or job-related features, or a combination is capable of predicting the mental health condition of clinicians or not.

## 2. Materials and Methods

**Figure 1:**
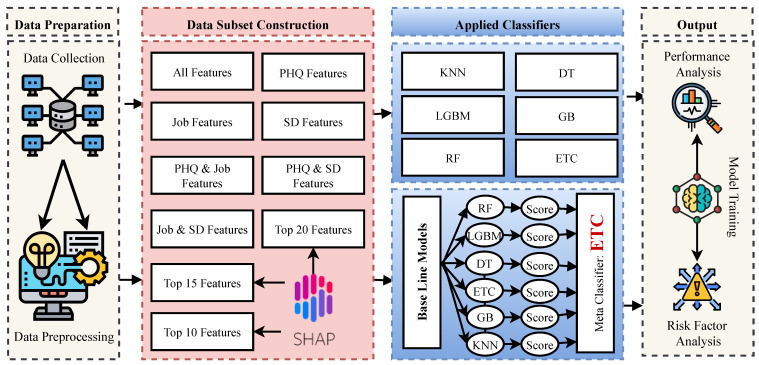
Overall framework of the study.

In this study, Google Colaboratory, a Python environment, was employed to analyze the data and build the predictive model to predict the depression levels of physicians. To build the predictive model and investigate the important risk factors, a dataset was built collecting information from doctors in Dhaka city. Then, the dataset was prepared as necessary to make it compatible with machine learning models. The dataset was then divided into different sub-categories to identify the best-fit sub-category for building the predictive models and identifying the significant risk factors. In this study, six traditional machine learning models were applied, and another stacking classifier was built using these algorithms to find a highly efficient model to predict the depression level of physicians with higher accuracy. All the procedures and applied methods are illustrated in Figure 1 sequentially. The following subsections of this section demonstrate all the applied techniques used in this study.

### 2.1. Data Collection and Description

The dataset used in this study was collected from different public and private hospitals in Dhaka, which is the capital city of Bangladesh. All the participants in the study had completed their graduation from MBBS and BDS and were involved in the medical profession as physicians. To collect data, a self-administered questionnaire was designed, including socioeconomic status, level of depression, and job satisfaction of participants. Sociodemographic information of the participants was collected to find the socioeconomic status of the target group of participants, where 16 pieces of information were collected from each participant. Then, 9 different questions were added to the questionnaire to explore the level of depression based on the Public Health Questionnaire (PHQ-9) [30]. PHQ-9 has been verified for use in general practice for individuals with mental illnesses [31]. Every question contains four options to select, such as 0 (Not at all), 1 (Several days), 2 (More than half days), and 3 (Nearly every day). Then, the score is calculated, and the depression severity is found. Depression severity is categorized following the sum of the score, where 0–4 indicates none, 5–9 mild, 10–14 moderate, 15–19 moderately severe, and 20–27 severe. It has also been found from the Satisfaction of Employees in Health Care (SEHC) that job-related issues are also significantly correlated to depression [32]. Therefore, 18 questions were added to the questionnaire for the assessment of the SEHC of each participant. Each SEHC question contains four options to choose such as 1 (Strongly disagree), 2 (Disagree), 3 (Agree), and 4 (Strongly agree). The SEHC total score is determined by averaging the 18 items, with higher scores reflecting better satisfaction. In addition to these, two other questions were added to the questionnaire related to smoking and daily exercise. A total of 45 questions were considered to collect data from medical professionals to determine the level of depression. The well-designed questionnaire was sent to 380 medical professionals, and 325 responses were collected.

After collecting data from participants, Body Mass Index (BMI) was calculated for each patient. According to the WHO BMI cut-off, the BMI was computed using the participants’ self-reported height and weight. Underweight was defined as having a body mass index (BMI) of less than 18.5. A BMI of 18.5 to 23 used to be considered a healthy weight range. Overweight and obese were defined as having a BMI of 23.0 to 25.0 and 25.0 or above, respectively [33].

### 2.2. Data Preprocessing

Data preprocessing is an obligatory task for obtaining an optimal result and performance of a machine learning model. Mainly, data preprocessing is performed on unrefined datasets to increase the prediction capability. In this phase, missing values are usually handled, but no missing values were found in the collected dataset. Data cleaning was performed where irrelevant features were removed. Generally, all numerical values are considered numeric types of features in the dataset. Therefore, the data type was defined for all the categorical features because all the categories were defined by numeric values. Then, it was found that the collected dataset was imbalanced, which resulted in poor model performance and an inefficient model. The dataset was balanced using Synthetic Minority Oversampling Technique (SMOTE).

### 2.3. Model Interpretation for Feature Selection

Model interpretation is one of the most important tasks to identify the reason for providing a correct or incorrect prediction by a machine learning model. It is also important to figure out how the prediction was formed and what role the specific features had in predicting the outcome. Model interpretability can assist to identify the feature importance and impact of each class on a specific feature. Since the identification of the most appropriate features is the most significant task for building an efficient machine learning model, the model interpretation technique was employed to calculate the feature importance score and rank them accordingly. In this technique, the feature importance score can be calculated for a single instance and the entire dataset as well. The training dataset was applied to estimate the feature importance score for feature selection in this study. The SHapley Additive exPlanations (SHAP) approach was used for model interpretation to show feature impact on each class and for feature selection. SHAP employs well-known game theory principles and a local explanation approach to evaluate the degree to which each feature contributes to the model’s overall decision-making abilities [34,35]. The following equation is used to determine SHAP values by using numerous axioms to apportion the contribution of each feature for a dataset containing *N* features and f(N) target features to predict [36].

(1) $$\emptyset_{i}=\sum_{S\subseteq N_{i}}^{}\frac{|S|!(K-|S|-1)!}{K!}\left[f\left(S\cup i\right)-f\left(S\right)\right]$$

Here $\emptyset_{i}$ refers to the feature importance of ith attribute to predict the expected output of the model, and it is assigned based on their marginal contribution. The number of independent features is denoted by *K*, and *S* represents the set of non-zero indexes in $z^{\prime }$. A fast SHAP estimation method, Tree Explainer, was employed for RF, LGB, GB, and DT models to identify the significant features [37].

In this study, five different feature subsets were formed, splitting the main dataset based on the category of features and denoted as DB1, DB2, DB3, DB4, and DB5. DB1, DB2, and DB3 were formed based on PHQ-9, sociodemographic, and job-related features, respectively. DB4 and DB5 were formed by top 20 and top 15 features employing SHAP value, also known as shapely value.

### 2.4. Supervised Machine Learning Model

In this study, seven types of classification algorithms were applied to build the predictive model for identifying the level of depression and significant risk factors. The applied classifiers are described in brief in this subsection.

#### 2.4.1. K Nearest Neighbor (KNN)

K Nearest Neighbor (KNN) is a type of supervised machine learning algorithm applied to building both classification and regression models. It is also known as a lazy learner method since it does not train from the training set immediately; instead, it keeps the dataset in run-time memory and then interacts with it during classification [38]. It identifies a test instance depending on the closeness. This means that when new metadata is presented, the KNN technique can swiftly classify it into a suitable group. The method compares an instance’s attributes to those of previously classified examples and estimates how similar the characteristics are. The class with the lowest characteristic similarity is then chosen as the instance’s class. Because it frequently examines more than one neighbor for identification of its class, it is known as KNN, where *k* is the number of points considered for classification. It is a challenging task for KNN to decide the optimal value of *k* [39]. KNN is applied for nonlinear data. KNN is very easy to understand and gives higher accuracy, but it is computationally expensive since it requires runtime memory to load the previously used data. In this study, the optimal value of *k* was 3 for the used datasets.

#### 2.4.2. Decision Tree (DT)

A decision tree (DT) is one of the oldest and most supervised machine learning approaches, which is used for solving both classification and regression-related tasks. To decide the output of the classification problem, a tree is built. Similar to a tree, the built tree also has a root node, some leaf nodes, and a decision node. The root node, also known as the parent node, refers to the starting point of the tree, and every node derived from the root node is known as the leaf node or terminal mode. The final node, which refers to the output node, is called the decision node. For deciding the root node and other subcategory nodes are selected based on feature importance. Which feature carries the highest importance value is considered as the root node, and then based on the feature importance value, the features are given priority to build the tree. For calculating feature importance values, different techniques are considered, including Information Gain (IG), Gini index (GI), Gain Ratio (GR), Reduction in Variance (RV), and Chi-Square (CS) [40]. IG is used for categorical features, and GI is used for continuous features. Entropy is calculated for IG and GI values following the below equation [41]:

(2) $$E\left(S\right)=\sum_{i=1}^{c}-p_{i}\log_{2}p_{i}$$

Here, E(S) represents the entropy of the current node, where $p_{i}$ represents the probability of an event known as *i*. Then the IG is calculated following the equation below [41]:

(3) IG=Entropybeforesplitting−Entropyaftersplitting

After calculating the IG, GR is calculated following the equation mentioned below [42]:

(4) $$GR=\frac{IG}{Entropy}$$

GI, RV, and CS are calculated following the equations mentioned below [41,42]:

(5) $$GI=1-\sum_{i=1}^{c}{\left(p_{i}\right)}^{2}$$

(6) $$RV=\frac{\sum {\left(x-\bar{x}\right)}^{2}}{n}$$

(7) $$CS=\sum \frac{{\left(O-E\right)}^{2}}{E}$$

Here $p_{i}$ refers to the probability of *i*th instance, *x* refers to the actual value, and *n* refers to the number of values where $\bar{x}$ represents the mean of all values. In addition, *O* and *E* represent the observed and expected score of the selected feature.

#### 2.4.3. Gradient Boosting (GB)

One of the most effective methods in machine learning is the gradient boosting (GB) technique. It is a version of ensemble techniques in which numerous weak models are created and combined to improve overall performance. This signifies that a set of separate models leads to a final model. The model is constructed in different stages. Individual models have low prediction power and over-fitting issues, but the ensemble of these models produces better results by controlling the overfitting issue [43]. Individual models in the ensemble are not generated on fully random selections of training data, but rather by giving greater weight to the incorrectly predicted data. The errors of ML algorithms are generally categorized into two types: bias errors and variance errors. As one of the boosting strategies, gradient boosting is used to decrease the bias error of GB [44]. It can be applied to both regression and classification issues. The cost function in regression problems is MSE, whereas the cost function in classification issues is Log-Loss. GB is a well-performed and mostly used ML algorithm.

#### 2.4.4. LightGBM (LGBM)

LGBM stands for Light Gradient Boosting Method, which is mostly known as LightGBM. It is an architecture for gradient boosting that employs tree-based training methods. It is intended to be dispersed and effective, with the following benefits [44]:

- Increased training pace and effectiveness.
- Reduce memory utilization.
- Increased precision.
- Parallel, distributed, and GPU learning are all supported.
- Capable of managing enormous amounts of data

It employs two innovative techniques: Gradient-based One Side Sampling and Exclusive Feature Bundling (EFB), which overcome the restrictions of the histogram-based approach employed in all GBDT (Gradient Boosting Decision Tree) frameworks [43]. The properties of the LightGBM Algorithm are formed by the two methodologies of GOSS and EFB explained below. They work together to make the model operate efficiently and to provide it a competitive advantage over alternative GBDT architectures.

#### 2.4.5. Random Forest (RF)

A random forest is an ensemble and meta-predictor that employs averaging to increase predicted accuracy and manage over-fitting by fitting a collection of decision tree classification models on different sub-samples of the dataset [40]. Instead of depending on a single decision tree, the random forest collects the results from each tree and predicts the ultimate result based on the majority vote of predictions. The larger number of trees in the forest results in higher accuracy and controls the overfitting issue. RF takes less and also is able to handle large amounts of data with high dimensionality to gain higher accuracy efficiently. Another advantage of RF is that it can handle datasets with missing values [45]. Although RF can be used for both classification and regression tasks, it is mostly used for classification and gains higher accuracy compared to other traditional machine learning algorithms.

#### 2.4.6. Extra Tree Classifier (ETC)

Extra tree classifier (ETC) is a form of ensemble learning approach that combines the classification results of numerous de-correlated decision trees aggregated in a “forest” [46]. In general, it is extremely similar to a Random Forest Classifier and differs mainly in the way the decision trees in the forest are constructed. The Extra Trees Forest’s Decision Trees are built from the initial training data. Then, at each test node, each tree is given a random sample of k features from the feature set, from which each decision tree must choose the best feature to partition the data using some mathematical criterion (typically the Gini Index) [47]. This random selection of characteristics results in the construction of numerous de-correlated decision trees. Based on the value of the Gini Index, feature importance is calculated, and then an optimized tree is built, which results in the optimized forest. Therefore, the prediction result of this algorithm is highly accurate and also controls the overfitting issue.

#### 2.4.7. Stacking Classifier (StackDPP)

**Figure 2:**
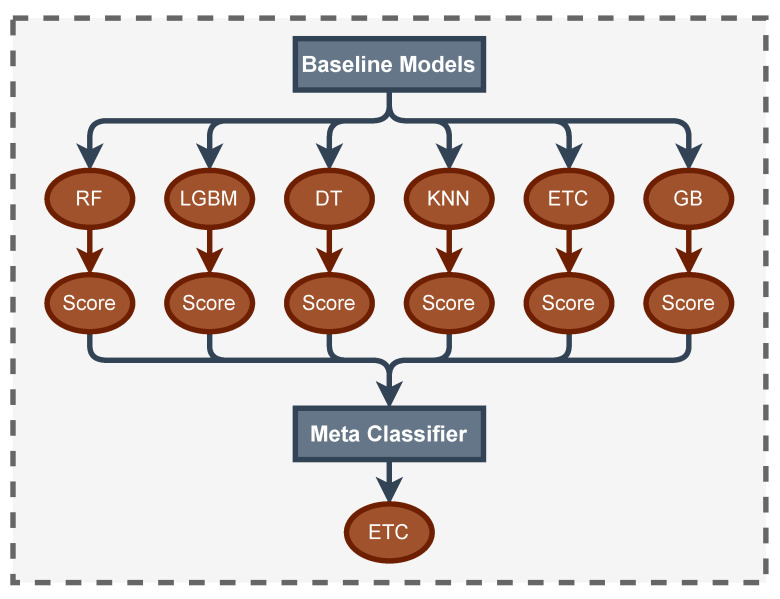
Structural Architecture of Proposed Model (StackDPP).

StackDPP is our proposed stacking-based ensemble classifier. Stacking is a technique for assembling classification or regression algorithms that use a two-layer estimation method [48]. The first layer is made up of some of the classification or regression algorithms which are known as baseline models. Baseline models are used to predict the output on the test datasets. The second layer comprises a single and final classifier or regression algorithm, which is known as Meta-Classifier or Regressor. Meta classifier or regressor accepts all of the baseline model predictions as input and generates new predictions. Combining the multiple algorithms and two-stage prediction results stacking-based predictive model gains higher accuracy compared to traditional machine learning models [49]. The structural architecture of StackDPP is depicted in Figure 2.

### 2.5. Performance Evaluation Metrics

The performance evaluation of a classification algorithm is one of the most significant tasks in the field of machine learning. In this study, six different evaluation metrics were used to evaluate performance. Based on these evaluation metrics, the performances of all the applied classifiers were compared with each other in order to find the best-performing classification. A brief overview of all the performance evaluation metrics is represented in Table 1.

## 3. Experimental Results Analysis

To conduct this study, the Python programming language was employed to apply different preprocessing techniques, exploratory data analysis (EDA), machine learning classifiers, and other approaches. Six different traditional methods were applied in this study: K Nearest Neighbor (KNN), Decision Tree (DT), Light Gradient Boosted Machine (LGBM), Gradient Boosting (GB), Random Forest (RF), and Extra Tree Classifier (ETC). Finally, considering these classifiers as baseline models, a stacking classifier was built for higher accuracy and efficiency to predict the depression level. To train and test the models, a 10-fold cross-validation technique was used. The following subsection represents the results and findings of the study.

### 3.1. Result of Exploratory Data Analysis

**Figure 3:**
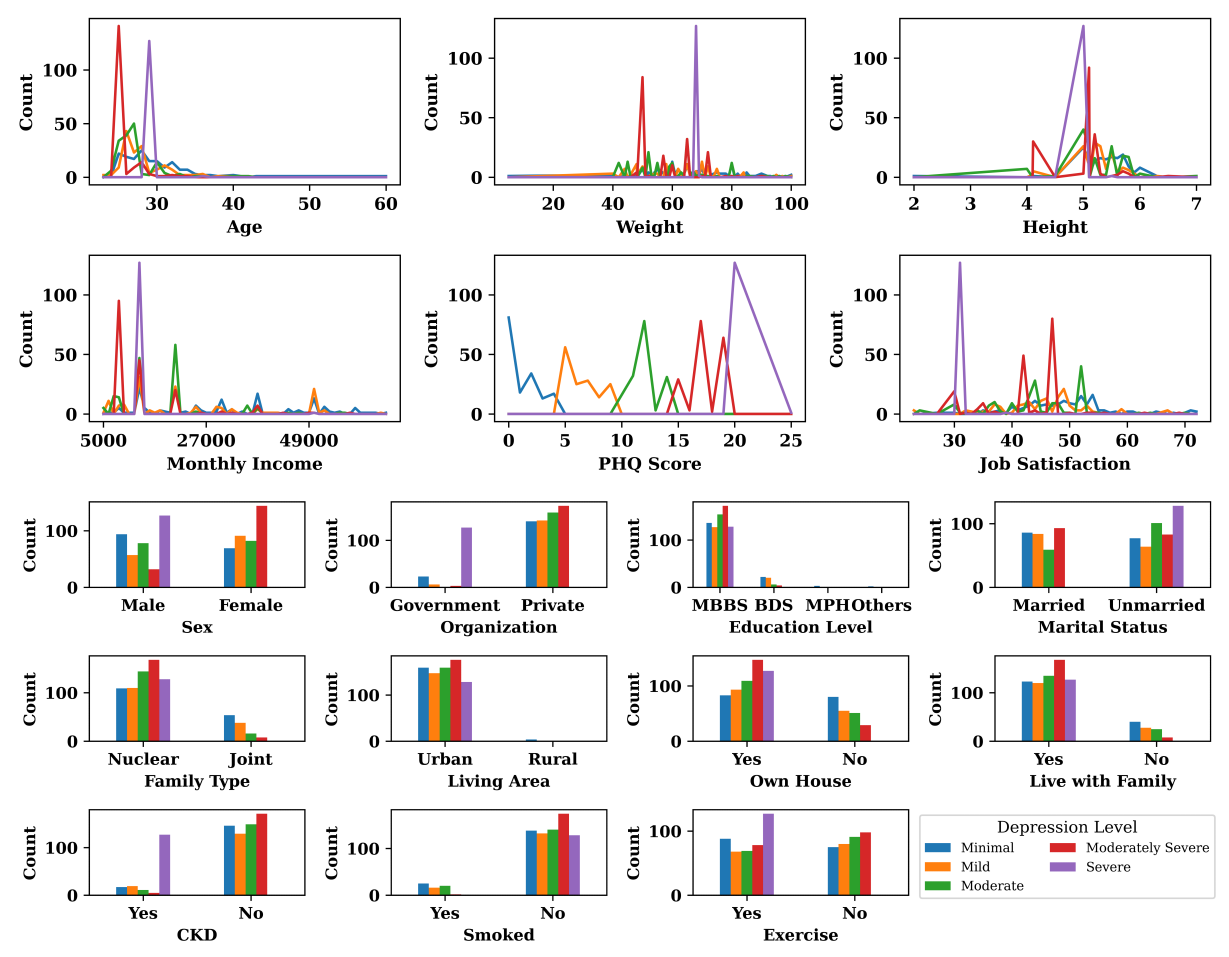
Result and explanation of exploratory data analysis for all the features.

At this stage, exploratory data analysis (EDA) was performed to find patterns and hidden knowledge in the dataset. The result of EDA is represented in Figure 3.

According to the results shown in Figure 3, it was found that most of the young (Age ≤30) and unmarried physicians were experiencing moderately severe and severe depression. It was also found that higher weight, lower monthly personal income, and lower job satisfaction were also major risk factors for depression among physicians. Figure 3 also reveals that male physicians experienced more severe depression than female physicians, whereas female physicians mostly experienced moderately severe depression. The physicians who served in private organizations were more mentally depressed than government service holders. In addition to that, it was found that physicians who lived in urban areas and lived in a nuclear family were more mentally depressed than physicians who lived in rural areas and lived in a joint family. Having a chronic disease was also another risk factor for depression among physicians.

### 3.2. Result of Supervised Machine Learning

#### 3.2.1. Performance Analysis for All the Features

Firstly, the whole dataset was preprocessed as necessary to make it compatible with the machine learning classifiers. Then, all the selected classifiers, including the stacking (StackDPP) classifier, were applied to the processed dataset, and the performances of the applied classifiers are represented in Table 2. Table 2 represents the 10-fold cross-validation results of all the applied methods, which were trained to employ all the features. Table 2 demonstrates that KNN gained the least accuracy with 0.748387 accuracy. In the score of all the performance measurement metrics, the performance of KNN at 0.956129, is not satisfactory. RF and ETC produced satisfactory values of 0.945806 and 0.956129 accuracy value. The findings of Table 2 represent that the proposed StackDPP method outperformed with 0.962581 accuracy, precision, recall, and f1 score. Therefore, the proposed method’s performance is really satisfactory.

#### 3.2.2. Performance Analysis for PHQ-Related Features

After the evaluation of all the applied methods with all the features, the models were evaluated using only PHQ-related features to predict the level of depression among physicians, and the performance results are demonstrated in Table 3. The table shows that DT produced lower performance compared to other applied classifiers, although other classifiers, except ETC and StackDPP, produced similar performance. However, ETC and StackDPP showed the same and highest performance with 0.923871 accuracy, precision, recall, f1 score, 0.904592 MCC, and 0.904579 kappa statistics values. In the score of PHQ-related features, it was found that the proposed StackDPP method outperformed the others.

#### 3.2.3. Performance Analysis for Sociodemographic Features

When the models were trained using only sociodemographic features to predict the depression level, it was found that KNN performed the worst with 0.699355 accuracy, precision, recall, and f1 score. The best performance was noticed in LGBM, which has 0.816774 accuracy, precision, recall, f1 score, 0.771119 MCC, and 0.770388 kappa statistic value. The StackDPP method also performed close to LGBM in terms of sociodemographic features. All the performance results of all the models for sociodemographic features are represented in Table 4.

#### 3.2.4. Performance Analysis for Job-Related Features

All the selected models were applied to job-related features only to predict the level of depression among physicians, and the performance of the models is represented in Table 5. It is found from the table that the performance of all the models was not satisfactory in terms of job-related features since other features play a more important role in the applied models. The highest accuracy was found at 0.792258, which was not good enough for predicting such an important issue as depression. Only job-related features were not good enough to predict the level of depression among clinicians and physicians.

#### 3.2.5. Performance Analysis for PHQ and Job-Related Features

Thereafter, the features related to PHQ and job were taken into consideration to evaluate models, and the results are presented in Table 6. The table shows that DT produced a lower performance of 0.895484 accuracy compared to other applied classifiers, although other classifiers gained more than 90% accuracy. ETC and StackDPP showed the same and the highest performance in terms of accuracy, precision-recall, and f1 score with 0.948387 values. However, in terms of MCC and kappa statistics, ETC and StackDPP produced 0.935689 and 0.935327 MCC, respectively, and 0.93534 and 0.935315 kappa statistics, respectively. In terms of PHQ and job-related features, it was found that the proposed StackDPP method outperforms other applied methods.

#### 3.2.6. Performance Analysis for PHQ and Sociodemographic Features

Another subset was formed by combining the PHQ and sociodemographic features. The performance result of the subset of datasets is represented in Table 7 for all the applied classification algorithms. It is found in Table 7 that KNN provided the least performance, while GB, RF, and ETC attained performances close to each other. However, the proposed classifier, StackDPP, outperformed with 0.947097 accuracy compared to all the applied classification algorithms.

#### 3.2.7. Performance Analysis for Job and Sociodemographic Features

Another subset of the dataset was constructed, combining the job and sociodemographic features. Then all the models are applied to this subset of data, and their performances are represented in Table 8. KNN and DT produced the worst performance for the dataset, while LGBM and RF gained higher performance compared to KNN and DT. Though ETC and StackDPP methods attained the same accuracy, precision, recall, and f1 score, StackDPP showed better MCC and kappa statistics than ETC. Therefore, the StackDPP method outperformed with 0.852903 accuracy for the job and sociodemographic features.

#### 3.2.8. Performance Analysis for the Selected Features

Shapely Additive Explanations (SHAP) techniques were applied to the models for all feature datasets and selected the top 20 features responsible for predicting the level of depression among physicians and clinicians. Then three individual subsets are constructed using these top 20 features: Top 20, Top 15, and Top 10. Then all the models were applied to these three selected sub-datasets, and their performances are represented in Table 9. From Table 9, it is found that the subset, constructed by the top 20 features, outperformed other subsets, such as the top 10 and top 15, with 0.96129 accuracy by StackDPP.

#### 3.2.9. Overall Performance Analysis of Machine Learning Models

**Figure 4:**
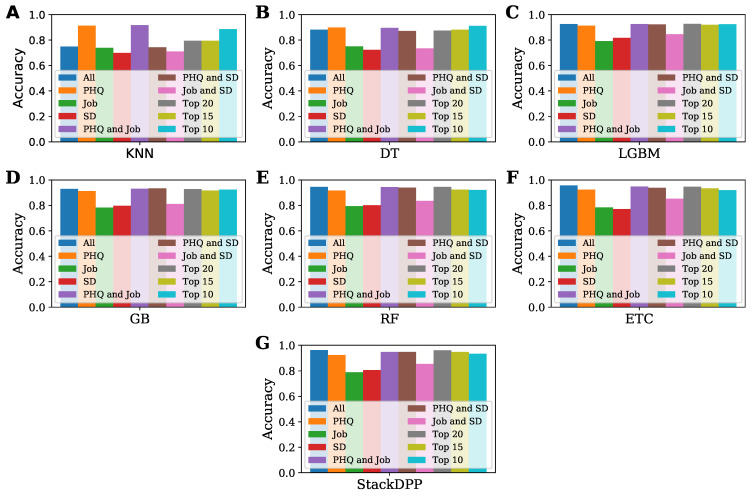
Performance comparison among all the sub-datasets for all the applied classifiers. (**A**) KNN; (**B**) DT; (**C**) LGBM; (**D**) GB; (**E**) RF; (**F**) ETC; (**G**) StackDPP.

The study aimed to find the best classifier to predict the level of depression of a physician and the best possible group of features that have the highest potential to determine the level of depression in a physician. To fulfill our objective, 10 subsets were formed, including the total dataset, and seven classifiers were employed, where one classification model was proposed, named after StackDPP. The overall performance of all the classifiers and the subset of data is presented in this subsection to find out the best group of features based on the accuracy of the applied classifiers and the best classifier for each subset of data. The performances of all the sub-datasets have been compared with each other and represented in Figure 3. At the same time, the performance of all the applied classifiers for each subset of datasets has been compared and is represented in Figure 4 for a better understanding of the performance of both the dataset and classifier.

The performances of all the sub-datasets for each classifier are represented in Figure 4. The figure illustrates that the subset formed by the combination of job and SD-related features and the subset formed by only job and SD-related features are not good enough to predict depression levels and that they are not greatly responsible for depression among physicians; rather, there are more attributes that are also greatly responsible for depression among physicians. KNN gained the most accuracy with the subset formed by only PHQ-related features and the subset formed by combining PHQ and job-related features. For all the classifiers except KNN, the subsets formed by all the features, PHQ, PHQ and job, PHQ and SD, and the top-selected features by SHAP value can be considered the best subset. To be more exact, the subset with all the features played a vital role in gaining the highest accuracy (0.962581), while the top 20 features selected from all the features based on SHAP value also gained the highest accuracy (0.96129), which is close to the dataset with all the features. Therefore, it cannot be said that only one type of feature is responsible for depression among physicians. All types of features are responsible for depression among physicians, more or less.

**Figure 5:**
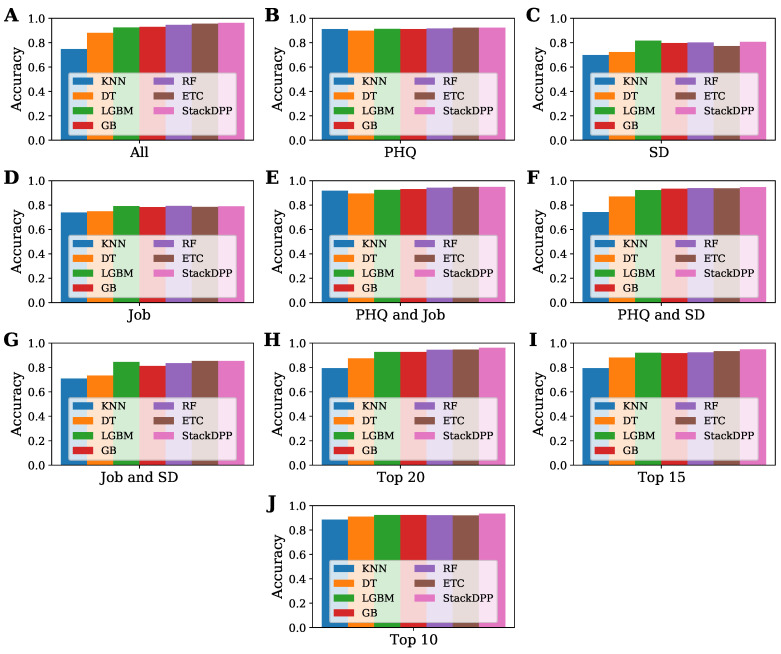
Performance comparison among all the applied classifiers for all the sub-datasets. (**A**) All; (**B**) PHQ; (**C**) SD; (**D**) Job; (**E**) PHQ and Job; (**F**) PHQ and SD; (**G**) Job and SD; (**H**) Top 20; (**I**) TOP 15; (**J**) Top 10.

The performance of all the classifiers for each dataset is represented and compared in Figure 5. According to the figure, KNN gained the least accuracy for all the datasets, while DT gained the least accuracy for the subsets formed by PHQ, and combining both PHQ and job-related features. The proposed algorithm, StackDPP, outperformed all the other algorithms in terms of accuracy for all the subsets. The figure indicates that the proposed classifier, StackDPP, is highly capable of predicting the level of depression and finding the most significant risk factors for physicians.

#### 3.2.10. Identification of Important Risk Factors for Mental Health

**Figure 6:**
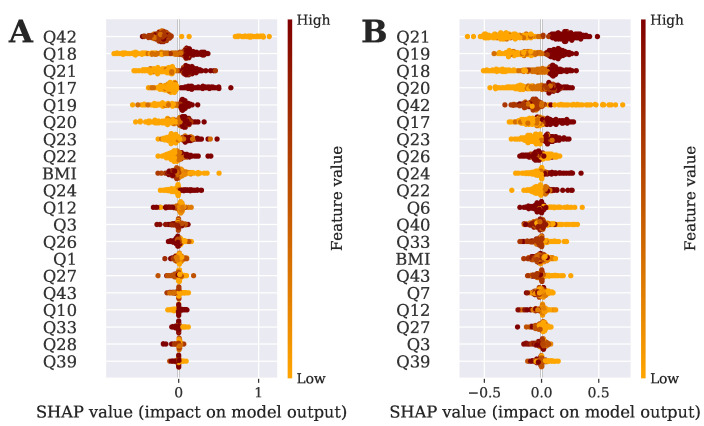

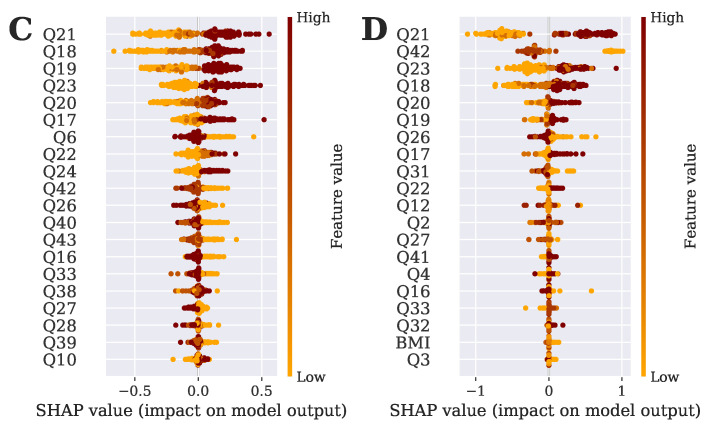
Summary plot of SHAP illustrating 20 most significant risk factors with feature impacts on depression level prediction. (**A**) 20 most significant risk factors found by GB; (**B**) 20 most significant risk factors found by RF; (**C**) 20 most significant risk factors found by ETC; (**D**) 20 most significant risk factors found by DT.

Finding the significant risk factors of any disorder or disease is an important task since treatment and therapy depend on the attributes most responsible for the disease. It is the same for mental health as well. The treatment, therapy, or other initiative will depend on the attributes most responsible for depression. Therefore, the top 20 significant risk factors are also found in this study. In addition to that, our proposed model also identifies the most significant risk factor. The risk factors, found in this study using the SHAP technique are represented in Figure 6. It is found in the figure that job-related issues and PHQ-related issues are highly responsible for the depression of physicians.

## 4. Discussion

The mental condition of physicians plays a vital role in keeping a nation fit and healthy. Therefore, it is crucial to identify the level of depression and related attributes which are responsible for depression in a physician. Some recent studies have shown that job satisfaction and job-related issues are mainly responsible for medical professionals, such as nurses and doctors [28]. Therefore, we included job-related issues along with other attributes, such as PHQ, sociodemographic, and personal information. The dataset was constructed considering all of these issues so that the most significant and accurate risk factors are found. The study mainly focuses on building an automatic predictive model with the best possible set of attributes to predict the level of depression of a physician considering different types of attributes and finding the most relevant and significant risk factors.

In this study, the dataset was divided into 10 different sub-datasets to identify the best possible set of attributes that are highly capable of predicting the level of depression among physicians. The sub-datasets were formed based on different categories of attributes. The categories of features are all the features, SD, PHQ, job, PHQ and job, PHQ and SD, job and SD, top 20 features, top 15 features, and top 10 features. Each category of features represents an individual sub-dataset. The top 20, 15, and 10 features are selected based on SHAP value using applied classifiers. Then, seven different classification algorithms were applied to all the sub-datasets, and compared the testing result of all the classifiers and sub-datasets based on accuracy, precision, recall, f1 score, MCC, and kappa statistics. The applied classifiers are KNN. DT, LGBM, GB, RF, ETC, and StackDPP. StackDPP is a stacking-based ensemble classifier which is proposed by this study. The architecture is represented in Figure 2. The study found that StackDPP is outperforming all the datasets and the best-performing sub-dataset is the dataset with all the attributes and the top 20 features. The StackDPP with the sub-dataset with all the attributes gained the highest accuracy (0.962581) and the top 20 attributes are enough to gain 0.96129 accuracy by StackDPP which is close to the performance of all the attributes. If the computational cost and run-time are considered, StackDPP is highly capable of predicting the level of depression of a physician with the top 20 attributes. Therefore, the proposed model, StackDPP, is highly capable of predicting depression with the top 20 attributes.

In addition to that, all the risk factors were analyzed based on the SHAP value and represented in Figure 5. It was found from the risk factor analysis that job-related issues are highly responsible for depression even though PHQ-related issues are also responsible. The top reason for depression among physicians is poor appetite or overeating and bad collaboration among colleagues. In addition, other attributes are also found in Figure 5. The details of the questions can be found in Supplementary Materials. Overall, the results and findings indicate that the proposed model, StackDPP, is highly capable of predicting the level of depression among physicians with the top 20 features with less computational cost. Otherwise, StackDPP can be applied to all the attributes for higher accuracy.

## 5. Conclusions

Depression is a mental health condition that affects both mental and physical health. Because physicians are the most important stakeholder of a nation, keeping them mentally healthy is crucial to building a healthy nation. From that perspective, this study proposed a stacking-based ensemble classifier, which is known as StackDPP. The proposed model, StackDPP, is highly capable of predicting the level of depression in physicians according to the dataset with all the attributes with 0.962581 accuracy and by the sub-dataset with the top 20 attributes with 0.96129 accuracy. In addition to that, the proposed model, StackDPP, is able to reveal the most significant risk factors. The proposed model will enable psychiatrists to diagnose depression and analyze the risk factors. The model also will contribute to increasing awareness among physicians to keep them mentally healthy by identifying risk factors. The proposed model has a limitation, which is time complexity. To reduce time complexity, in the future, we will build an advanced deep learning-based ensemble model to diagnose mental health conditions.

## Figures and Tables

**Table 1:** Brief description of performance evaluation metrics.

Evaluation Criteria	Explanation	Formula
Accuracy	Accuracy is the ratio of correctly classified instances [48].	$Acc=\frac{TP+TN}{TP+TN+FP+FN}$
Precision	Precision is a valid assessment parameter when we need to be highly confident in our forecast. Precision is defined as the ratio of True Positives to all Positives [49].	$Precision=\frac{TP}{TP+FP}$
Recall	The recall is a test of how well our model identifies True Positives [50].	$Recall=\frac{TP}{TP+FN}$
F-Measure	F1 Score is the weighted average of Precision and Recall [51].	$F=\frac{2\;\times \;Precisioon\;\times \;Recall}{Precsion+Recall}$
Kappa Statistics	It evaluates the performance of qualitative characteristics from expected and observed inter-rater interaction [51].	$K_{p}=1-\frac{1-p_{o}}{1-p_{e}}$
MCC	It is essentially a correlation coefficient number ranging from −1 to +1 [50].	$MCC=\frac{TP\;\times \;TN-FP\;\times \;FN}{\sqrt{\left(TP+FP)(TP+FN)(TN+FP)(TN+FN\right)}}$

**Table 2:** Performance evaluation of all the applied classifiers for all the features.

Classifiers	Accuracy	MCC	Kappa	Precision	Recall	F1
KNN	0.748387	0.686697	0.684685	0.748387	0.748387	0.748387
DT	0.881290	0.851288	0.851226	0.881290	0.881290	0.881290
LGBM	0.92516	0.906331	0.906206	0.925161	0.925161	0.925161
GB	0.930323	0.912740	0.912674	0.930323	0.930323	0.930323
RF	0.945806	0.932326	0.932116	0.945806	0.945806	0.945806
ETC	0.956129	0.945075	0.945012	0.956129	0.956129	0.956129
StackDPP	0.962581	0.953152	0.953087	0.962581	0.962581	0.962581

**Table 3:** Performance evaluation of all the applied classifiers for PHQ-related features.

Classifiers	Accuracy	MCC	Kappa	Precision	Recall	F1
KNN	0.912258	0.890218	0.890033	0.912258	0.912258	0.912258
DT	0.898065	0.87239	0.872206	0.898065	0.898065	0.898065
LGBM	0.913548	0.891663	0.89164	0.913548	0.913548	0.913548
GB	0.912258	0.890166	0.89006	0.912258	0.912258	0.912258
RF	0.917419	0.896591	0.896487	0.917419	0.917419	0.917419
ETC	0.923871	0.904603	0.904571	0.923871	0.923871	0.923871
StackDPP	0.923871	0.904592	0.904579	0.923871	0.923871	0.923871

**Table 4:** Performance evaluation of all the applied classifiers for sociodemographic features.

Classifiers	Accuracy	MCC	Kappa	Precision	Recall	F1
KNN	0.699355	0.624561	0.623198	0.699355	0.699355	0.699355
DT	0.722581	0.652358	0.652291	0.722581	0.722581	0.722581
LGBM	0.816774	0.771119	0.770388	0.816774	0.816774	0.816774
GB	0.797419	0.74657	0.746212	0.797419	0.797419	0.797419
RF	0.80129	0.751976	0.750981	0.80129	0.80129	0.80129
ETC	0.771613	0.714728	0.713827	0.771613	0.771613	0.771613
StackDPP	0.806452	0.761496	0.757392	0.806452	0.806452	0.806452

**Table 5:** Performance evaluation of all the applied classifiers for job-related features.

Classifiers	Accuracy	MCC	Kappa	Precision	Recall	F1
KNN	0.739355	0.676446	0.673519	0.739355	0.739355	0.739355
DT	0.749677	0.686364	0.686093	0.749677	0.749677	0.749677
LGBM	0.792258	0.739725	0.739466	0.792258	0.792258	0.792258
GB	0.783226	0.728512	0.728228	0.783226	0.783226	0.783226
RF	0.793548	0.742098	0.741251	0.793548	0.793548	0.793548
ETC	0.784516	0.730979	0.73007	0.784516	0.784516	0.784516
StackDPP	0.789677	0.73649	0.736337	0.789677	0.789677	0.789677

**Table 6:** Performance evaluation of all the applied classifiers for PHQ and job-related features.

Classifiers	Accuracy	MCC	Kappa	Precision	Recall	F1
KNN	0.917419	0.896684	0.896503	0.917419	0.917419	0.917419
DT	0.895484	0.869003	0.868998	0.895484	0.895484	0.895484
LGBM	0.925161	0.906401	0.906248	0.925161	0.925161	0.925161
GB	0.931613	0.914344	0.9143	0.931613	0.931613	0.931613
RF	0.943226	0.929018	0.928859	0.943226	0.943226	0.943226
ETC	0.948387	0.935689	0.93534	0.948387	0.948387	0.948387
StackDPP	0.948387	0.935327	0.935315	0.948387	0.948387	0.948387

**Table 7:** Performance evaluation of all the applied classifiers for PHQ and sociodemographic features.

Classifiers	Accuracy	MCC	Kappa	Precision	Recall	F1
KNN	0.743226	0.681195	0.678136	0.743226	0.743226	0.743226
DT	0.870968	0.838398	0.838313	0.870968	0.870968	0.870968
LGBM	0.922581	0.903034	0.902983	0.922581	0.922581	0.922581
GB	0.934194	0.917665	0.917557	0.934194	0.934194	0.934194
RF	0.939355	0.924123	0.923994	0.939355	0.939355	0.939355
ETC	0.938065	0.922468	0.922383	0.938065	0.938065	0.938065
StackDPP	0.947097	0.933839	0.93367	0.947097	0.947097	0.947097

**Table 8:** Performance evaluation of all the applied classifiers for job and sociodemographic features.

Classifiers	Accuracy	MCC	Kappa	Precision	Recall	F1
KNN	0.709677	0.637304	0.635947	0.709677	0.709677	0.709677
DT	0.734194	0.667581	0.667125	0.734194	0.734194	0.734194
LGBM	0.845161	0.806528	0.805889	0.845161	0.845161	0.845161
GB	0.811613	0.764333	0.763889	0.811613	0.811613	0.811613
RF	0.834839	0.793837	0.793101	0.834839	0.834839	0.834839
ETC	0.852903	0.816465	0.815654	0.852903	0.852903	0.852903
StackDPP	0.852903	0.821431	0.815536	0.852903	0.852903	0.852903

**Table 9:** Performance evaluation of all the applied classifiers for the selected features.

Number of Selected Features	Classifiers	Accuracy	MCC	Kappa	Precision	Recall	F1
Top 20	KNN	0.794839	0.744718	0.742895	0.794839	0.794839	0.794839
	DT	0.874839	0.843509	0.843148	0.874839	0.874839	0.874839
	LGBM	0.926452	0.907929	0.907851	0.926452	0.926452	0.926452
	GB	0.927742	0.90952	0.909452	0.927742	0.927742	0.927742
	RF	0.945806	0.93226	0.9321	0.945806	0.945806	0.945806
	ETC	0.947097	0.933922	0.933709	0.947097	0.947097	0.947097
	StackDPP	0.96129	0.951558	0.951472	0.96129	0.96129	0.96129
Top 15	KNN	0.794839	0.744524	0.742907	0.794839	0.794839	0.794839
	DT	0.88129	0.851396	0.851252	0.88129	0.88129	0.88129
	LGBM	0.92	0.89979	0.899739	0.92	0.92	0.92
	GB	0.917419	0.89674	0.896538	0.917419	0.917419	0.917419
	RF	0.923871	0.904849	0.904619	0.923871	0.923871	0.923871
	ETC	0.934194	0.917771	0.917547	0.934194	0.934194	0.934194
	StackDPP	0.948387	0.9354	0.935281	0.948387	0.948387	0.948387
Top 10	KNN	0.886452	0.858205	0.857725	0.886452	0.886452	0.886452
	DT	0.910968	0.888464	0.888414	0.910968	0.910968	0.910968
	LGBM	0.923871	0.904685	0.904613	0.923871	0.923871	0.923871
	GB	0.923871	0.904748	0.904591	0.923871	0.923871	0.923871
	RF	0.92129	0.901525	0.90138	0.92129	0.92129	0.92129
	ETC	0.92	0.899887	0.899754	0.92	0.92	0.92
	StackDPP	0.934194	0.917578	0.917509	0.934194	0.934194	0.934194

## Data Availability

On reasonable request, the datasets are available from the corresponding author.

## Supplementary Materials

The following supporting information can be downloaded at: https://www.mdpi.com/article/10.3390/bioengineering10070858/s1.
