# Supplementary material for: StackDPP: Stacking-Based Explainable Classifier for Depression Prediction and Finding the Risk Factors among Clinicians
Source: Bioengineering (Basel). 2023 Jul 20;10(7):858. doi: 10.3390/bioengineering10070858 (PMC10376085; doi:10.3390/bioengineering10070858)
Supplement: Supplementary file 1 [file bioengineering-10-00858-s001.zip › bioengineering-2483250-supplementary.pdf]

Questionnaire

Depression among medical professionals in Bangladesh

Serial no: [ ]

A| Socio-demographic Questions

1. Age____________ years
2. Weight___________kg
3. Height______________Ft.
4. Sex: ①Male ②Female
5. What is your occupation/Designation? ______________
6. What's type of organization are you working? ①Government ② Non-government ③Self
7. How many year ago have you finished your Bachelor/Diploma courses? _____________
8. What is your highest level of education? ①MBBS ②BDS③DIploma④BSc ⑤MPH ⑥Others____
9. Religion:①Muslim ②Hindu ③Buddhist ④Christian ⑤Others
10. Marital status : ①Married ②Unmarried
11. Types of family: ① Nuclear ② Joint
12. Monthly personal income (Approximately) _________________tk.
13. Where you have been living for last two years?①Urban area ②Rural area
14. Do you live in your own house? ①Yes ②No
15. Do you live with your family? ①Yes ②No
16. Are you suffering from any kinds of chronic diseases? ①Yes ②No.If Yes, Which _________

B| Depression Assessment

17. Little interest or pleasure in doing things?
    ⓪Not at all ① several days ②More than half of the days ③Nearly every days
18. Feeling down, depressed, or hopeless?
    ⓪Not at all ① several days ②More than half of the days ③Nearly every days
19. Trouble falling or staying asleep, or sleeping too much?
    ⓪Not at all ① several days ②More than half of the days ③Nearly every days
20. Feeling tired or having little energy?
    ⓪Not at all ① several days ②More than half of the days ③Nearly every days
21. Poor appetite or overeating?
    ⓪Not at all ① several days ②More than half of the days ③Nearly every days
22. Feeling bad about yourself — or that you are a failure or have let yourself or your family down?
    ⓪Not at all ① several days ②More than half of the days ③Nearly every days
23. Trouble concentrating on things, such as reading the newspaper or watching television?
    ⓪Not at all ① several days ②More than half of the days ③Nearly every days
24. Moving or speaking so slowly that other people could have noticed? Or so fidgety or restless that you have been moving a lot more than usual?
    ⓪Not at all ① several days ②More than half of the days ③Nearly every days

25. Thoughts that you would be better off dead, or thoughts of hurting yourself in some way?
⓪Not at all ① several days ②More than half of the days ③Nearly every days

C| Job satisfaction analysis

	**Strongly Disagree**	**Disagree**	**Agree**	**Strongly Agree**
26. The management of this organization is supportive of me.	①	②	③	④
27. I receive the right amount of support and guidance from my direct supervisor.	①	②	③	④
28. I am provided with all trainings necessary for me to perform my job.	①	②	③	④
29. I have learned many new job skills in this position.	①	②	③	④
30. I feel encouraged by my supervisor to offer suggestions and improvements.	①	②	③	④
31. The management makes changes based on my suggestions and feedback.	①	②	③	④
32. I am appropriately recognized when I perform well at my regular work duties.	①	②	③	④
33. The organization rules make it easy for me to do a good job.	①	②	③	④
34. I am satisfied with my chances for promotion.	①	②	③	④
35. I have adequate opportunities to develop my professional skills.	①	②	③	④
36. I have an accurate written job description.	①	②	③	④
37. The amount of work I am expected to finish each week is reasonable.	①	②	③	④
38. My work assignments are always clearly explained to me.	①	②	③	④
39. My work is evaluated based on a fair system of performance standards.	①	②	③	④
40. My department provides all the equipment, supplies, and resources necessary for me to perform my duties.	①	②	③	④
41. The buildings, grounds, and layout of this facility are adequate for me to perform my duties.	①	②	③	④
42. My coworkers and I work well together.	①	②	③	④
43. I feel I can easily communicate with members from all levels of this organization.	①	②	③	④

D| Others

44. Do you smoke? ①Yes ②No
45. Do you walk/exercise every day at least 15 minute apart from your regular activities?①Yes②No
